# Recombinant GI-19 infectious bronchitis virus induction of humoral immunosuppression with reduced HI antibody response following Newcastle disease virus vaccination and bursal cell apoptosis

**DOI:** 10.1186/s13567-026-01839-2

**Published:** 2026-09-24

**Authors:** Chenyan Wang, Weijia Zhang, Bo Hou

**Affiliations:** Institute of Animal Husbandry and Veterinary Medicine, Fujian Academy of Agricultural Sciences/Fujian Animal Disease Control Technology Development Center/Fujian Key Laboratory for Control and Prevention of Avian Diseases, Fuzhou, Fujian 350013 People’s Republic of China

**Keywords:** Infectious bronchitis virus, immunosuppression, bursa of Fabricius, apoptosis, tropism

## Abstract

Pathological changes and viral presence in the bursa of Fabricius (BF) have been reported, but the immunosuppression of infectious bronchitis virus (IBV) remains poorly characterized. Here, we systematically evaluated the pathogenicity of two GI-19 lineage IBV isolates, CK/CH/FJ-B3/2023 (bursal origin) and CK/CH/FJ-T5/2023 (tracheal origin), in 7-day-old specific pathogen-free (SPF) chicks, including further assessments of serum cytokines and the bursal apoptosis. We further assessed the impacts of these two IBV isolates on the Newcastle disease virus (NDV) inactivated vaccine-induced hemagglutination inhibition (HI) antibody response. This study reveals that both GI-19 lineage IBV isolates, which originated from the recombination of the 4/91 vaccine strain, exhibit bursa-tropism, inducing bursal pathological atrophy, prolonged viral detection [up to 35 days post-infection (dpi)] and delayed seroconversion until 14 dpi. Notably, IBV infection induces humoral immunosuppression in SPF chickens, evidenced by a ≥ 1log_2_ reduction in NDV-HI antibody titers at 14 and 28 dpi, with antibody production recovering to normal levels by 35 dpi. This is accompanied by a sustained systemic proinflammatory response characterized by persistent upregulation of tumor necrosis factor-*α* (TNF-*α*), interleukin 1*β* (IL-1*β*), IL-6, and interferon-gamma (IFN-*γ*) in the serum of infected chicks up to 28 dpi. Early-onset bursal apoptosis was detected as early as 1 dpi, with the bursal apoptosis index significantly higher than that of the negative control throughout the observation period. These findings highlight the humoral immunosuppression induced by IBV variants. Further characterization of IBV types is warranted to elucidate IBV immunosuppressive mechanisms.

**Supplementary information:**

The online version contains Supplementary Material available at https://doi.org/10.1186/s13567-026-01839-2.

## Introduction

Since its first identification in 1936 [1], the infectious bronchitis virus (IBV) has emerged as one of the most prevalent respiratory pathogens in the commercial poultry industry. It primarily replicates in the respiratory tract, but subsequent studies confirmed its presence in nonrespiratory tissues [2], including the kidney, proventriculus, oviduct, duodenum, jejunum, and rectum, as well as immune organs such as the bursa of Fabricius (BF), spleen, Harderian gland (HG), and cecal tonsils (CT) [3, 4], possibly due to blood circulation [5]. Viral persistence in immune organs varied in duration, with frequent detection in the BF or CT [4, 6].

As a gammacoronavirus, IBV undergoes rapid evolution under selection pressure in chickens, driven by frequent mutations and recombination events that lead to the emergence of new genotypes or variants [7]. Currently classified IBV genotypes cover GI to GIX [8–10], encompassing 38 viral lineages with most demonstrating multiorgan tropism [11, 12]. Certain isolates, such as the DMV/1639 variants initially characterized as nephropathogenic, have also been found to infect the reproductive tract [13], oviduct [14], and lymphoid organs [15]. Different serotypes or genotypes of IBV exhibit varying pathogenic abilities, even within the same genotype. For example, QX isolates from different countries (Chinese, French, Slovakian, and Greek) show differences in pathogenicity and affinity for certain tissues [16], with obvious histological changes found in trachea, kidney, and oviduct [16, 17].

Immunosuppressive agents such as infectious bursal disease virus (IBDV) [18] and chicken anemia virus (CAV) [19] can rapidly replicate in lymphoid organs and induce apoptosis [20, 21]. Coinfection with IBV and these immunosuppressants delays Immunoglobulin A/Immunoglobulin G (IgA/IgG) production, hinders growth, and prolongs viral persistence [22, 23]. Previous research showed that chicks seroconverted by 14 days post-infection (dpi) of IBV [11], but the impact on the immune responses to other vaccines is unclear. Although IBV has been isolated in immune tissues, its involvement in functional damage such as apoptosis of immune cells remains undetermined. At the early phase of IBV infection, the increase in proinflammatory responses plays a key role in activating immune responses, regulating inflammation, and mediating tissue damage. A different proinflammatory response was associated with IBV isolates of different genotypes [24] and specific genetic lines of chickens [25]. The highest upregulation of interleukin 6 (IL-6), interleukin 1β (IL-1*β*), and interferon-gamma (IFN-*γ*) in tissues coincided with microscopic lesions and the highest viral load [26]. Recent research indicates that the pathological lesions in the BF after IBV infection involve lymphoid follicle atrophy or depletion, with minimal lesions in the spleen and thymus [15, 27]. Regardless of the administered dose, viral RNA levels decline rapidly in chickens receiving a high dose of virus [28], and decreased IBV copies were detected in the BF at 28 dpi [29] and CT at 39 dpi [3]. It remains unclear whether prolonged detection of viral nucleic acids in immune tissues can cause persistent pathological damage and high-level expression of cytokines, ultimately leading to immunosuppression.

In this study, we isolated two IBV strains from BFs and tracheas. To determine whether IBV infection induces immunosuppression and impairs Newcastle disease virus (NDV) vaccine hemagglutination inhibition (HI) antibody production, we performed a comprehensive evaluation. This evaluation combined clinical signs, relative organ weights, tissue viral loads, cytokine detection, IBV and NDV-HI antibody detection, histopathology, bursal apoptosis, and immunohistochemistry examination.

## Materials and methods

### Virus isolation

Two IBV isolates were obtained from commercial broiler flocks located in Fujian Province, China, in May 2023 (Table 1). All birds had received immunization with live vaccines strains H120, 793/B-type, and QX-type at 1- and 14-days-old. The affected flocks exhibited clinical signs including respiratory distress and growth retardation, with corresponding gross pathological lesions. Tissue samples were collected from both clinically healthy and symptomatic chickens within these flocks. Notably, IBV remained detectable in the BFs even during the later stages of the production cycle.

**Table 1:** **The characteristics and origins of the two IBV isolates**

Viruses		Origin					Cross lesion
Identity	No. embryo passages before typical IB virus was observed	Type of bird	Age	Tissue	Positive rate of IBV	Positive rate of IBDV	
CK/CH/FJ-T5/2023	3	Broilers	24–26	Trachea	4/7^a^	–	Tracheal hemorrhage, airsacculitis, atrophy of the bursae, adenogastritis, and ventriculitis
CK/CH/FJ-B3/2023	5	Broilers	24–26	Bursa of Fabricius	40/40^b^	0/40	

^a^Positive chicken count/total tested (composite samples: five tissues per pool).

^b^Number of chickens testing positive/total tested.

Trachea and BF collection were performed as described previously, and five tracheal tissues were pooled for one sample [11]. After three to five blind passages in 10-day-old specific pathogen-free (SPF) embryos, IBV was detected either by dwarfing or curling of the embryos or by IBV testing of allantoic fluid. The allantoic fluid was screened with reverse transcription polymerase chain reaction (RT-PCR) or PCR for H9 avian influenza virus (AIV), *Mycoplasma synoviae* (MS), *Mycoplasma gallisepticum* (MG), NDV, CAV, IBDV, laryngotracheitis virus (LTV), avian leucosis virus (ALV), Marek’s disease virus (MDV), Reticuloendotheliosis virus (REV), Reovirus, and Adenovirus. The reverse transcription quantitative polymerase chain reaction (RT-qPCR) primers used for IBV detection were as previously described [28]. All isolates were purified by three limiting dilution passages as reported previously and stored at −70 ℃ [30]. The 50% embryo infectious dose (EID_50_) was calculated using the Reed–Muench method.

All SPF white leghorn chickens were hatched from fertile white leghorn SPF eggs purchased from Jinan Spafas Poultry Co., Ltd. (Jinan, China), and were housed in isolators.

### Sequencing, phylogenetic, and recombinant analysis

Extraction of viral RNA was performed using the QIAamp Viral RNA Mini Kit (Qiagen, Hilden, Germany), and whole genome sequence and recombination analyses were conducted as described previously [11].

Phylogenetic analysis was performed on the *S1* gene using 78 reference strains (Additional file 1) in MEGA version 7.0. Neighbor-Joining phylogenetic trees were assessed with 1000 bootstrap replicates.

### Pathogenicity experiments

All bird experiments were approved by the Institute of Animal Husbandry and Veterinary Medicine of the Fujian Academy of Agricultural Sciences and were performed in accordance with animal ethics guidelines and approved protocols. All husbandry procedures were conducted in compliance with the Animal Welfare Act and the Guide for the Care and Use of Laboratory Animals (permission no. MYLISC2024-015). Feed and water were provided ad libitum.

Experiment 1: 120 7-day-old SPF chicks were numbered and randomly divided into three groups—a negative control group (group 1), and two experimental groups challenged with 100 μL/bird eye drops with a dose of 10^5.5^ EID_50_ of CK/CH/FJ-T5/2023 (T5) (group 2) or CK/CH/FJ-B3/2023 (B3) isolate (group 3). The chicks were observed for clinical symptoms twice daily after inoculation up to 35 days. At 1, 3, 5, 7, 14, 21, 28, and 35 dpi, five chicks from each group were randomly removed, and blood was collected from the birds via wing venipuncture and tested for IBV antibodies. Additionally, blood samples at 1, 3, 5, 7, 14, and 28 dpi were subject to cytokine profiling to assess early immune responses. Cloacal swabs were also taken to detect IBV by RT-qPCR. Finally, all live chickens from each group were weighed, bled, and sacrificed by injection with tiletamine hydrochloride and zolazepam hydrochloride (Virbac, Carros, France). During necropsy, carcasses were examined for gross lesions. The thymus, spleen, and BF were excised and weighed, and portions of these organs, in addition to portions of the lower half of the trachea, were harvested and fixed immediately in 10% paraformaldehyde and followed by the evaluation of histopathologic lesions in the immune tissues. The remaining portions of these organs were stored at −70 ℃ for the viral load determination. Relative organ weights were calculated using the following formula: relative organ weight = [organ weight (g)/body weight (g)] × 100 [31].

Experiment 2: 30 7-day-old SPF chicks were randomly divided into three groups (groups 4, 5, and 6), and vaccinated with the NDV inactivated vaccine (Clone 30, Netherlands, MSD Co., Ltd) via subcutaneous injection in the neck at 0.2 mL/bird. Then, groups 5 and 6 were additionally inoculated with the same dose of the two IBV isolates (strain T5 and B3) via eye drops, respectively. Experiment 2 shared the same negative control group as experiment 1. NDV-HI antibody titers in the negative control group of experiment 1 were measured at 7, 14, 21, 28, and 35 dpi, while blood from G4, G5, and G6 was collected from each bird via wing venipuncture at 7, 10, 14, 21, 28, and 35 dpi and tested for IBV antibodies and NDV-HI antibody titers.

### IBV and NDV-HI antibody testing

An infectious bronchitis virus antibody test kit (IDEXX Laboratories, Westbrook, ME, USA) was used to detect serum anti-IBV antibodies, following the manufacturer’s instructions. An S:P ratio above 0.20 indicated positive results.

An NDV-HI test antigen, along with the NDV-negative and NDV-positive reference sera (LaSota, Yebio Co., Ltd, Qingdao, China) and 1% SPF chicken red blood cells (Hongquan Co., Ltd, Guangzhou, China), were used to determine the titer of NDV-specific HI antibody. The HI test was conducted using four HA units of antigen and read by tilting the plates at 60 ℃ for 1 min after an incubation period of 30 min at 20 ℃. Titers of 1/8 or more were regarded as positive.

### Proinflammatory cytokine bioassay

Chicken tumor necrosis factor-*α* (TNF-*α*), IL-1*β*, IL-6, and IFN-*γ* concentrations in serum were determined using commercial enzyme-linked immunosorbent assay (ELISA) kits (Meimian Industrial Co., Ltd., Jiangsu, China), according to the manufacturer’s protocols. The cytokines protein levels were quantified relative to a standard curve.

### RT-qPCR

Trachea, thymus, spleen, and BF samples were prepared and processed as previously described [30]. Viral RNA was extracted from the samples using the QIAamp Viral RNA Mini Kit (Qiagen, Hilden, Germany), and the RT-qPCR assay was performed as previously described. The IBV-specific primers (IBV 5′GU391 and IBV 5′GL533) and probes (IBV 5′G) were used as described in a previous report [28]. RT-qPCR was conducted on a Roche LightCycler 96 instrument. The 20 μL reaction mixture consisted of 4× Fast 1-Step Mix (Thermo Fisher Scientific, Vilnius, Lithuania), 0.80 μL of IBV-specific primers, 0.60 μL of probe, 5 μL of template RNA, and 7.8 μL of nuclease-free deionized water. For standard curve construction, a positive plasmid standard was serially diluted from 10^7^ to 10^2^ copies/μL. CT values were plotted against the log_10_ of copy numbers to generate the standard curve for quantification.

### Histopathology and immunohistochemistry

After a 48 h fixation, tissues were paraffin embedded, sectioned (5 μm), hematoxylin and eosin (H&E) stained, and histopathologically analyzed under a light microscope. The lesion scoring of the BF was revised based on previously published literature [32] and includes two indicators: bursal atrophy and inflammatory cell infiltration. The scoring criteria for the atrophy indicator are defined as follows: 0, normal; 1, minor depletion of lymphocytes in isolated follicles; 2, lymphocyte depletion in most of the follicles, hyperemia, and slight widening of interfollicular spaces; 3, cystic cavity formation in most follicles, vacuolation, and necrotic foci; and 4, severe depletion of lymphocytes in all follicles and marked widening of interfollicular spaces. The second indicator evaluates the degree of inflammatory cell infiltration using a 1–4 scoring scale. Score 1, minimal focal inflammatory cell infiltration; score 2, mild multifocal inflammatory cell infiltration; score 3, moderate diffuse inflammatory cell infiltration; and score 4, severe extensive inflammatory cell infiltration. For the spleen and thymus, lesion scoring is based on the degree of lymphocyte infiltration, using an identical 1–4 scale.

Five 28-day-old SPF chickens were inoculated with eye drops containing 100 μL of the B3 isolate at a dose of 10^5.5^ EID_50_ per bird. A booster challenge was administered after 2 weeks using the same dose and inoculation route. Blood was collected after another 14 days and separated for the collection of the hyperimmune serum for further use. The IBV in situ antigens were detected from the trachea with immunohistochemistry (IHC) staining and a monoclonal antibody targeting the N protein [11]. For IBV in situ antigen detection in the BF, spleen, and thymus, 5-µm tissue sections were antigen retrieved and incubated in 10% goat serum in PBS for 1 h to block nonspecific binding. Slides were then incubated with chicken anti-IBV hyperimmune serum at a 1:200 dilution in PBS for 4 ℃ overnight, followed by incubation with a horseradish peroxidase-conjugated goat antichicken IgG for 1 h. Sections were incubated with 3,3′-Diaminobenzidine (DAB, Sigma, St. Louis, MO, USA) for 15 min, counterstained with hematoxylin, air dried, and examined under a light microscope.

### TUNEL analysis

Paraffin-embedded bursal tissue sections were selected at 1, 3, 5, 7, 14, and 28 dpi, and a TUNEL assay was performed using an apoptosis detection kit (Servicebio Co., Ltd, Wuhan, China) to analyze apoptosis. Briefly, sections were deparaffinized and hydrated, followed by treatment with 20 µg/mL proteinase K at 37 ℃ for 20 min. The sections were then washed with 1 × equilibration buffer and incubated with Biotin-dUTP labeling mix. The TUNEL assay was then performed according to the manufacturer’s instructions. Positive cells were visualized using 3,3′-Diaminobenzidine (DAB) chromogen, and apoptotic cells were identified by their characteristic brown nuclear staining. For quantitative analysis, five sections were randomly selected from each sample under a light microscope, and positive cells were counted in five randomly selected fields per section. The apoptosis index (AI) was calculated using ImageJ 1.53e software (National Institutes of Health, Bethesda, MD, USA) using the following formula: AI = (TUNEL-positive cells/total number of cells) × 100%.

### Statistical analysis

All data were analyzed using GraphPad Prism version 10.0 (GraphPad, La Jolla, CA, USA). Changes in body weight, relative organ weights, and apoptosis index were analyzed with the Kruskal–Wallis test for nonparametric data, followed by Dunn’s multiple comparison test. Viral loads, NDV-HI antibody levels, and cytokine concentrations were analyzed using a two-way analysis of variance (ANOVA) with Bonferroni correction for multiple comparisons. Pathological lesion scoring was analyzed using the Mann–Whitney *U* test for nonparametric data. Results were regarded as significant at *P* < 0.05 (^*^), highly significant at *P* < 0.01 (^**^), extremely significant at *P* < 0.001 (^***^), and exceptionally significant at *P* < 0.0001 (^****^).

## Results

### Phylogenetic analysis of the *S1* gene

**Figure 1:**
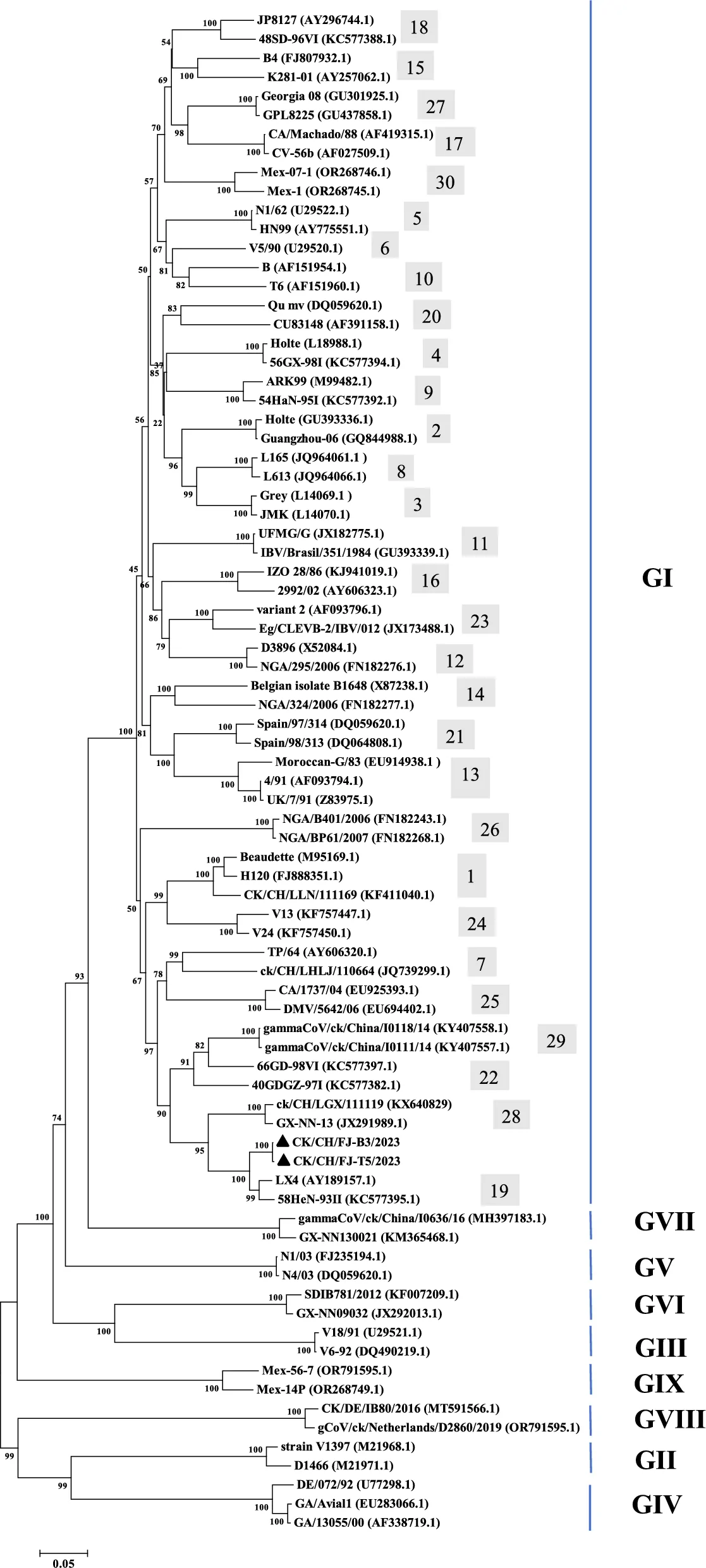
**Phylogenetic analyses of the *S1* gene (A) of the isolate CK/CH/FJ-B3/2023 (▲) and CK/CH/FJ-T5/2023 (▲) with other reference strains using the nearest Neighbor-Joining method with 1000 bootstrap replicates**

The complete genomic sequences of the B3 (bursal origin) and T5 (tracheal origin) IBV strains were deposited in GenBank (accession numbers PX103084 and PX103085). The 78 aligned reference genomes (Additional file 1) representing genotypes GI–GIX had a final length of 1517 bp. Evolutionary distances were computed using the Kimura two-parameter method, and the phylogenetic analysis of the complete *S1* gene sequences revealed that both strains clustered within the GI-19 lineage with nucleotide sequence similarities of 94.69–95.13% (B3) and 95.19–95.50% (T5) with the GI-19 genotype (Figure 1).

### Recombination analysis of IBV strains

**Figure 2:**
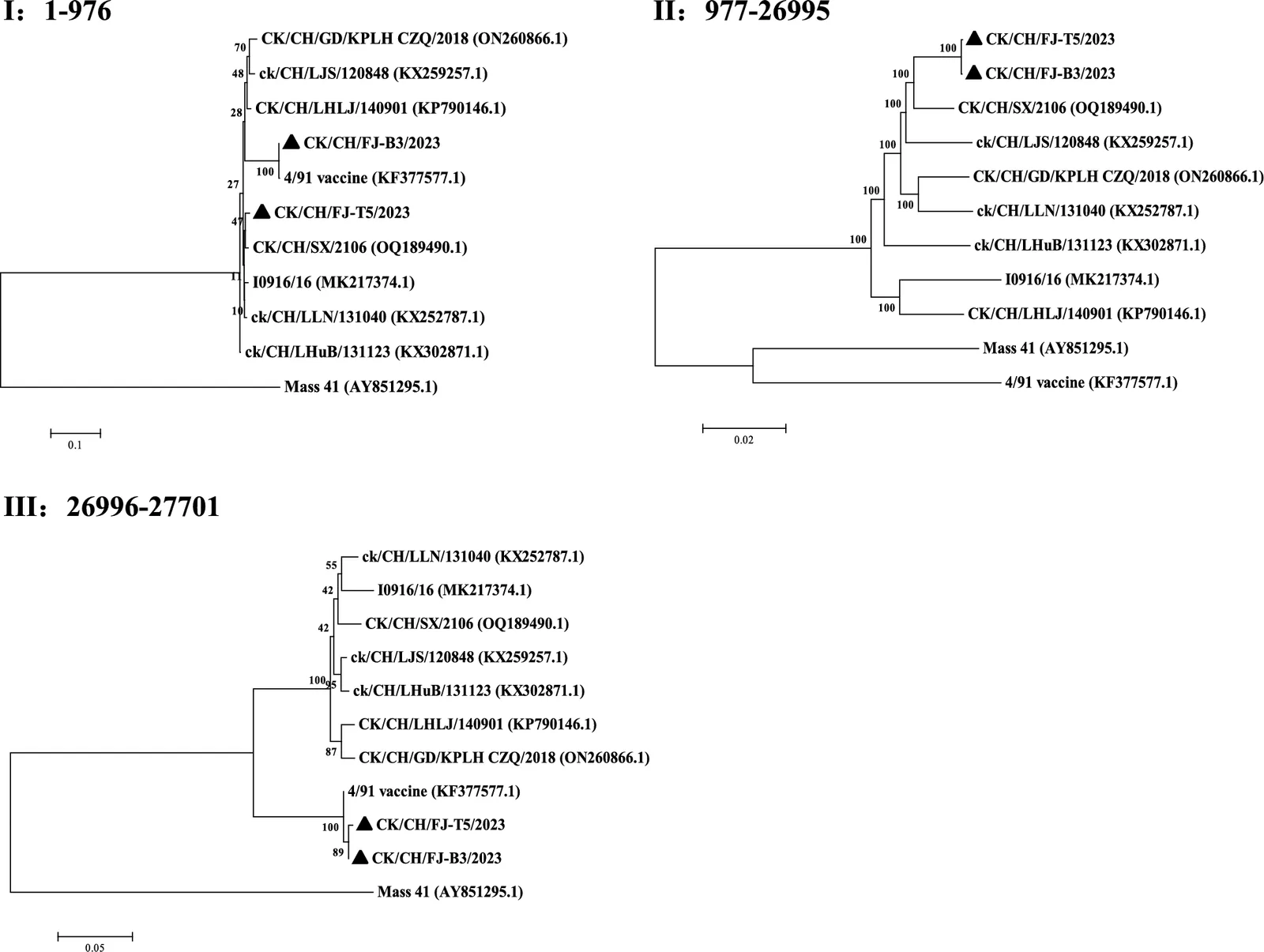
**Phylogenetic trees of different genome regions of CK/CH/FJ-B3/2023 (▲), CK/CH/FJ-T5/2023 (▲), CK/CH/SX/2106, the 4/91 vaccine, and other reference strains**

Recombination analysis in RDP4 revealed that both the B3 and T5 strains underwent recombination events with the 4/91 vaccine strain (KF377577.1) at distinct genomic locations. For strain B3, two recombination breakpoints were identified: one in the 5′-terminal region (nucleotides 1–976) and another in the 3′-terminal region (nucleotide 26,996 to the end of the genome). Strain T5 had a single recombination event restricted to the 3′-terminal region (nucleotides 26,950 to the end of the genome). Analysis confirmed that CK/CH/SX/2106 (OQ189490.1) was the major parental strain, and the 4/91 vaccine strain was the minor parental contributor. Phylogenetic analysis further confirmed these relationships among these IBV strains (Table 2; Figure 2).

**Table 2:** **Recombinant breakpoints, genes, and major and minor sequences of genomic recombination events of CK/CH/FJ-B3/2023 and CK/CH/FJ-T5/2023 strains**

Isolate	Breakpoints		Genes	Major sequence	Minor sequence	Detection method (*P*-value)
	Start	End				
CK/CH/FJ-B3/2023	1	976	*1ab*	CK/CH/SX/2106 (OQ189490.1)	4/91 vaccine (KF377577.1)	7.020 × 10^–81^ (RDP), 1.907 × 10^–85^ (GENECONV), 1.558 × 10^–17^ (MaxChi), 1.441×10^–17^ (Chimaera), 2.885 × 10^–20^ (SiScan), 6.217 × 10^–14^ (3Seq)
	26,996	–	*N*	CK/CH/SX/2106 (OQ189490.1)	4/91 vaccine (KF377577.1)	1.447 × 10^–86^ (RDP), 6.778 × 10^–86^ (GENECONV), 2.327 × 10^–19^ (MaxChi), 4.269 × 10^–20^ (Chimaera), 4.130 × 10^–30^ (SiScan), 9.447 × 10^–06^ (3Seq)
CK/CH/FJ-T5/2023	26,950	–	*N*	CK/CH/SX/2106 (OQ189490.1)	4/91 vaccine (KF377577.1)	2.499 × 10^–87^ (RDP), 1.089 × 10^–86^ (GENECONV), 6.159 × 10^–19^ (MaxChi), 1.481 × 10^–18^ (Chimaera), 4.864 × 10^–30^ (SiScan), 5.792 × 10^–14^ (3Seq)

### Clinical signs

In the challenged groups of experiment 1, chicks were depressed, with respiratory symptoms (wheezing, coughing, head shaking, and tracheal rattles) as early as 3 dpi. Respiratory distress persisted for 13 days, and by 17 dpi, the chicks were huddled together, feather ruffling, particularly in the B3-challenged group. At 21 dpi, all the B3-challenged chicks were depressed with a significant reduction in feed intake, followed by the onset of diarrhea from 28 dpi that continued through 35 dpi. Throughout the experimental period, control groups maintained normal health without clinical signs.

### Gross lesions

**Figure 3:**
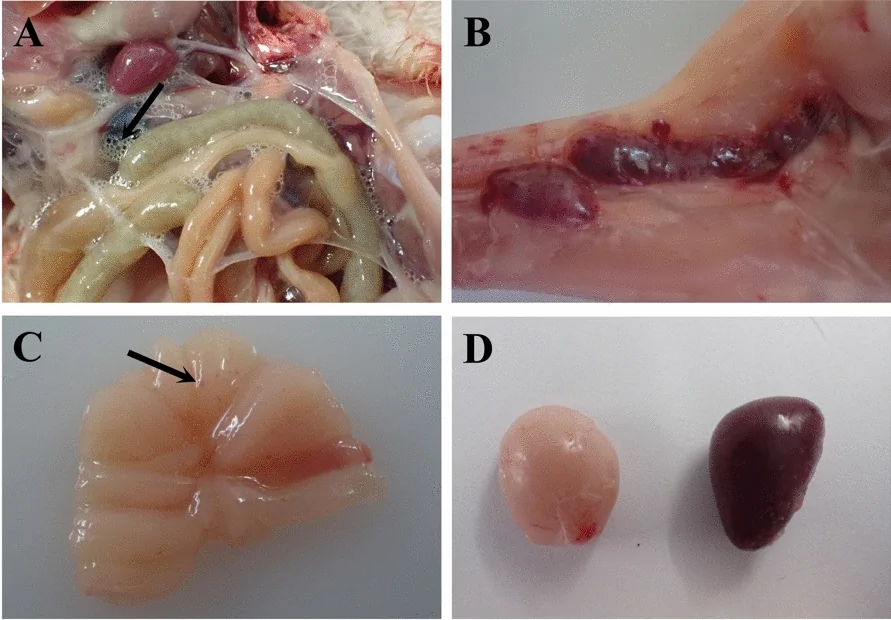
**Gross lesions in infected groups** **of experiment 1**. **A** Air-sac lesions of diseased chicken in 7-day-old infected groups [5 days post-infection (dpi)] (arrow). **B** Thymus with punctate hemorrhages in 7-day-old infected groups (3 dpi). **C** Bursa of Fabricius (BF) with punctate hemorrhages in 7-day-old infected groups (21 dpi) (arrow). **D** Atrophied BF (size comparable with the spleen) (28 dpi).

In experiment 1, necropsy revealed tracheal hemorrhages with excess mucus in 20% (1/5) of infected chicks at 14 and 21 dpi. The B3-challenged group exhibited early-onset airsacculitis at 3 dpi that persisted until 28 dpi, whereas the T5-challenged group showed airsacculitis from 7 to 21 dpi (Figure 3A). Notably, airsacculitis in both infected groups peaked at 14 dpi. Punctate thymic hemorrhages were found in infected groups, as early as 3 dpi (20%), followed by thymic enlargement at 5 dpi (20%), and thymic atrophy by 21 dpi (60% and 80% in T5- and B3-challenged groups, respectively) (Figure 3B). Of the T5-challenged group, 20% exhibited splenomegaly as early as 3 dpi, whereas the B3-challenged group showed enlargement at 14 dpi. A single case of splenic atrophy was found in the T5-challenged group at 14 dpi. Mucinous exudate appeared from the BF by 5 dpi, followed by hemorrhaging in infected chicks by 14 dpi (Figure 3C). The characteristic initial enlargement of the bursa was seen by 3 dpi, followed by progressive atrophy that started at 28 dpi, when the bursa was the same size as the spleen (Figure 3D). No obvious lesions were observed in the control group.

### ELISA and NDV-HI test

Seroconversion was initially detected in infected birds from experiment 1 at 14 or 21 dpi, while the IBV antibody seropositivity rate remained below 80% even at 35 dpi. Experiment 2 demonstrated similar ELISA results, with a maximum seroconversion rate of 70% (7 out of 10 tested birds) at 21 dpi in the B3-challenged group. Throughout the two experimental periods, all control group birds were seronegative for IBV (Table 3).

**Table 3:** **Results of the serology [IBV-enzyme-linked immunosorbent assay (ELISA) and NDV-hemagglutination inhibition (HI) antibody]**

Experiments	Antigen	Groups	dpi								
			1	3	5	7	10	14	21	28	35
1	IBV	G1	0/5^a^	0/5	0/5	0/5	–	0/5	0/5	0/5	0/5
		G2	0/5	0/5	0/5	0/5	–	0/5	3/5	2/5	2/5
		G3	0/5	0/5	0/5	0/5	–	1/5	1/5	3/5	2/5
2	IBV	G4	–	–	–	0/10	0/10	0/10	0/10	0/10	0/10
		G5	–	–	–	0/10	0/10	1/10	3/10	3/10	5/10
		G6	–	–	–	0/10	0/10	3/10	7/10	7/10	7/10
	NDV	G1	–	–	–	< 0	-	< 0	< 0	< 0	< 0
		G4	–	–	–	3.0 ± 0.0%^b^	3.0 ± 0.0%	5.8 ± 19.6%	6.4 ± 3.2%	7.8 ± 8.1%	7.4 ± 9.4%
		G5	–	–	–	3.0 ± 0.0%	3.0 ± 0.0%	4.1 ± 24.3%^***^	6.0 ± 11.1%	7.3 ± 9.2%	6.8 ± 9.3%
		G6	–	–	–	3.0 ± 0.0%	3.0 ± 0.0%	4.8 ± 25.6%	6.2 ± 19.8%	6.3 ± 19.9%^**^	7.0 ± 11.7%

^a^Number of chickens that seroconverted/number of chickens tested by ELISA.

^b^Mean ± coefficient of variation (CV) of NDV-HI.

^**^ and ^***^ compared with NDV-HI in G4, the results showed highly significant (*P* < 0.01) and extremely significant (*P* < 0.001).

In experiment 2, NDV HI antibody titers in the control group (G1) were below the limit of detection. The increase in HI antibody titers was relatively slow in the challenged groups, especially in G6. Specifically, at 14 dpi, the NDV-HI titers in chicks from G5 were extremely significantly lower than those of G4, showing a 1.7-log_2_ reduction. At 28 dpi, the NDV-HI titers in G6 chicks were highly significantly lower than those of G4, with a 1.5-log_2_ reduction (Table 3).

### Cytokines expression in serum

**Figure 4:**
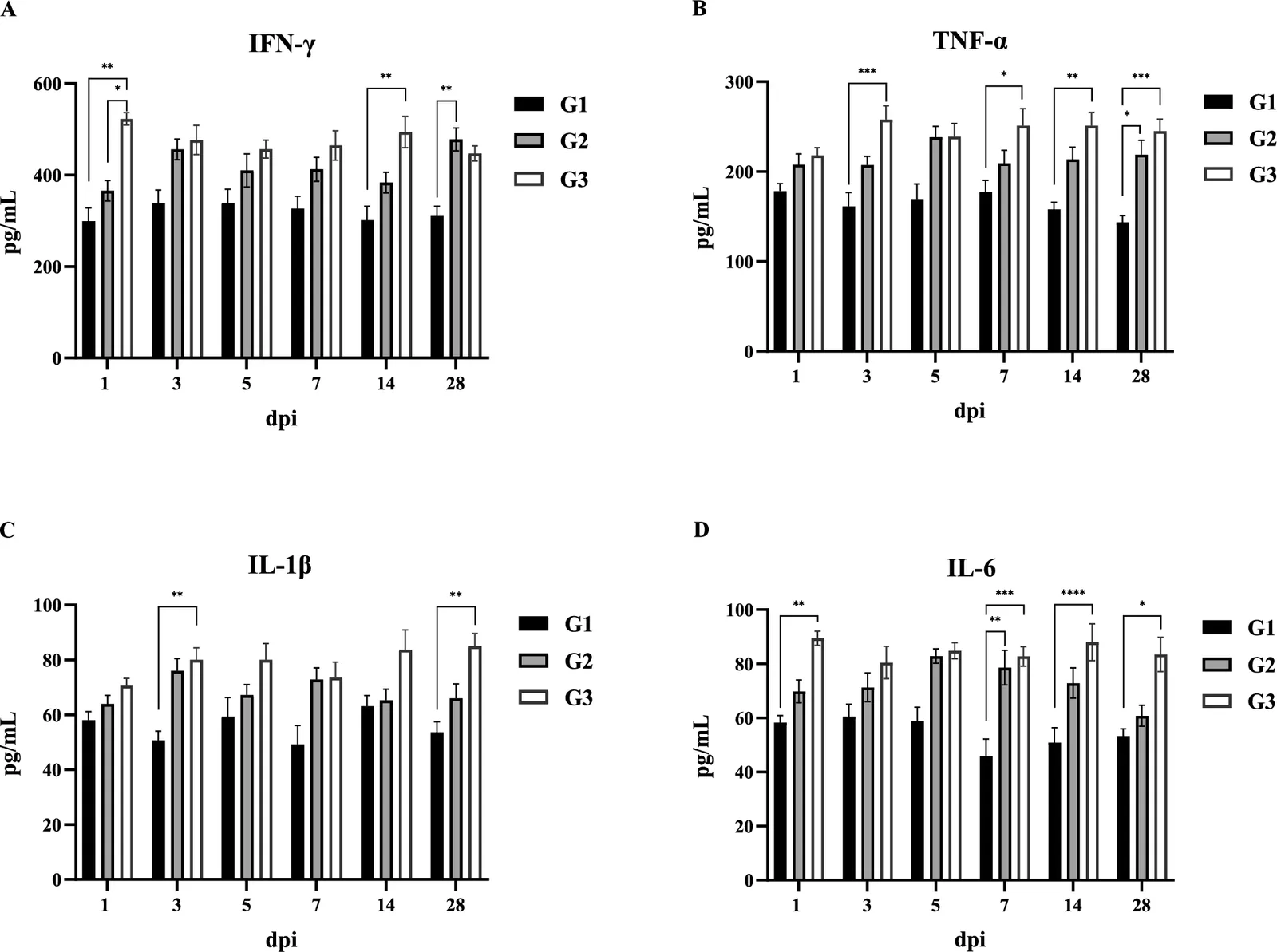
**Changes in the IFN-*γ*** **(A), TNF-*α* (B), IL-1*β* (C), and IL-6 (D) levels in the serum of 7-day-old SPF chickens in experiment 1**. The cytokines protein levels were quantified relative to a standard curve. Data were analyzed using two-way ANOVA, bars indicate the mean ± SEM.

The expression of the proinflammatory cytokines ‌IFN-*γ*, TNF-*α*, IL-1*β*, and IL-6 was upregulated in the serum of infected chicks at 1 dpi, and remained at elevated levels throughout the experimental period (Figure 4). All four cytokines were expressed at higher levels in the B3-challenged groups compared with the T5-challenged group, except for ‌IFN-*γ* at 28 dpi. Compared with the negative control group, the B3-challenged group showed significantly higher ‌IFN-γ levels at 1 and 14 dpi (*P* < 0.01), while the T5-challenged group only exhibited significantly higher ‌IFN-*γ* levels only at 28 dpi (*P* < 0.01) (Figure 4A). At 1 dpi, ‌IFN-*γ* levels in the B3-challenged group were significantly higher than those in the T5-challenged group (*P* < 0.05). For TNF-*α*, the B3-challenged group showed significantly elevated cytokine levels at 3, 7, 14, and 28 dpi compared with the negative control group, with significance ranging from *P* < 0.05 to *P* < 0.001. By contrast, the T5-challenged group only showed a significant increase at 28 dpi (*P* < 0.05) (Figure 4B). For IL-1*β*, the B3-challenged group exhibited significantly higher cytokine levels than the negative control group at 3 and 28 dpi (*P* < 0.01) (Figure 4C). For IL-6, the B3-challenged group showed significantly elevated levels at 1, 7, 14, and 28 dpi relative to the negative control group, with significance ranging from *P* < 0.05 to *P* < 0.0001, while the T5-challenged group only at 7 dpi showed a significant increase (*P* < 0.01) (Figure 4D).

### Relative organ weights

Compared with the negative control group, body weights decreased significantly in the T5- and B3-challenged group by 14 dpi (*P* < 0.05 and *P* < 0.01, respectively). A similar trend was observed in the B3-challenged group by both 28 and 35 dpi (*P* < 0.05 and *P* < 0.01, respectively). The B3-challenged group exhibited significantly increased spleen weight by 5 dpi (*P* < 0.05) but decreased by 35 dpi (*P* < 0.05), while the relative spleen weights increased significantly by both 3 and 5 dpi (*P* < 0.05 and *P* < 0.01, respectively). The BF weight of chickens in the B3-challenged group decreased significantly by 35 dpi (*P* < 0.05). Compared with the control group, the relative bursa weight in the B3-challenged group decreased by at least 0.13 at 28 and 35 dpi, whereas that in the T5-challenged group increased by 0.08 at 28 dpi (Table 4).

**Table 4:** **Body weight and relative organ weight (percentage of body weight) in 7-day-old groups at 1 to 35 dpi** **in experiment 1**

dpi	Groups	Body weight (g)	Thymus weight (g)	Thymus relative weight	Spleen weight (g)	Spleen relative weight	Bursa weight (g)	Bursa relative weight
1	G1	73 ± 0.05	0.31 ± 0.12	0.43 ± 0.15	0.06 ± 0.25	0.08 ± 0.22	0.22 ± 0.15	0.30 ± 0.14
	G2	69 ± 0.10	0.29 ± 0.30	0.42 ± 0.22	0.07 ± 0.25	0.09 ± 0.18	0.16 ± 0.20	0.24 ± 0.21
	G3	69 ± 0.08	0.25 ± 0.12	0.37 ± 0.17	0.07 ± 0.25	0.10 ± 0.23	0.18 ± 0.17	0.26 ± 0.11
3	G1	86 ± 0.05	0.39 ± 0.12	0.45 ± 0.15	0.08 ± 0.20	0.09 ± 0.18	0.24 ± 0.20	0.28 ± 0.15
	G2	81 ± 0.05	0.37 ± 0.19	0.45 ± 0.16	0.11 ± 0.32	0.13 ± 0.35	0.26 ± 0.14	0.32 ± 0.13
	G3	84 ± 0.04	0.36 ± 0.20	0.43 ± 0.18	0.10 ± 0.12	0.13 ± 0.12^a^	0.23 ± 0.17	0.28 ± 0.18
5	G1	95 ± 0.09	0.50 ± 0.33	0.51 ± 0.24	0.10 ± 0.21	0.11 ± 0.17	0.32 ± 0.20	0.33 ± 0.12
	G2	89 ± 0.18	0.41 ± 0.28	0.45 ± 0.14	0.12 ± 0.35	0.13 ± 0.26	0.25 ± 0.27	0.29 ± 0.24
	G3	98 ± 0.07	0.50 ± 0.21	0.51 ± 0.17	0.17 ± 0.17^a^	0.17 ± 0.11^A^	0.40 ± 0.23	0.40 ± 0.18
7	G1	118 ± 0.06	0.64 ± 0.14	0.54 ± 0.11	0.14 ± 0.17	0.12 ± 0.15	0.52 ± 0.25	0.44 ± 0.22
	G2	114 ± 0.12	0.53 ± 0.25	0.46 ± 0.18	0.20 ± 0.44	0.17 ± 0.35	0.54 ± 0.32	0.46 ± 0.20
	G3	113 ± 0.07	0.55 ± 0.22	0.48 ± 0.19	0.19 ± 0.26	0.16 ± 0.26	0.47 ± 0.16	0.42 ± 0.20
14	G1	211 ± 0.05	1.26 ± 0.22	0.60 ± 0.21	0.31 ± 0.23	0.15 ± 0.26	0.98 ± 0.21	0.46 ± 0.17
	G2	185 ± 0.05^a^	0.98 ± 0.19	0.53 ± 0.14	0.26 ± 0.22	0.14 ± 0.21	0.83 ± 0.37	0.45 ± 0.38
	G3	174 ± 0.12^A^	0.95 ± 0.37	0.53 ± 0.32	0.28 ± 0.31	0.16 ± 0.25	0.84 ± 0.27	0.48 ± 0.17
21	G1	293 ± 0.09	2.19 ± 0.18	0.75 ± 0.18	0.46 ± 0.17	0.16 ± 0.15	1.54 ± 0.22	0.52 ± 0.17
	G2	264 ± 0.20	1.70 ± 0.37	0.62 ± 0.22	0.46 ± 0.28	0.18 ± 0.18	1.21 ± 0.31	0.45 ± 0.16
	G3	245 ± 0.24	1.54 ± 0.34	0.61 ± 0.16	0.36 ± 0.27	0.15 ± 0.15	1.20 ± 0.25	0.49 ± 0.07
28	G1	438 ± 0.07	3.12 ± 0.33	0.71 ± 0.30	0.81 ± 0.10	0.19 ± 0.11	1.87 ± 0.29	0.42 ± 0.23
	G2	380 ± 0.12	2.15 ± 0.15	0.57 ± 0.15	0.80 ± 0.26	0.21 ± 0.25	1.87 ± 0.18	0.50 ± 0.21
	G3	347 ± 0.11^a^	1.65 ± 0.41	0.46 ± 0.31	0.83 ± 0.29	0.24 ± 0.25	1.04 ± 0.30	0.30 ± 0.26
35	G1	577 ± 0.10	4.43 ± 0.44	0.76 ± 0.39	1.27 ± 0.17	0.22 ± 0.14	3.22 ± 0.26	0.56 ± 0.24
	G2	534 ± 0.08	3.05 ± 0.45	0.57 ± 0.43	1.14 ± 0.32	0.21 ± 0.26	2.69 ± 0.23	0.50 ± 0.17
	G3	425 ± 0.08^A^	2.72 ± 0.22	0.64 ± 0.19	0.83 ± 0.08^a^	0.19 ± 0.07	1.81 ± 0.23^A^	0.43 ± 0.27

Means calculated form biruds per groups. Means within a column and a time point with different lowercase superscripts are significantly different compared with the negative group (*P* < 0.05), and means within a column and a time point with different uppercase superscripts are significantly different compared with the negative group (*P* < 0.01).

### Viral load

**Figure 5:**
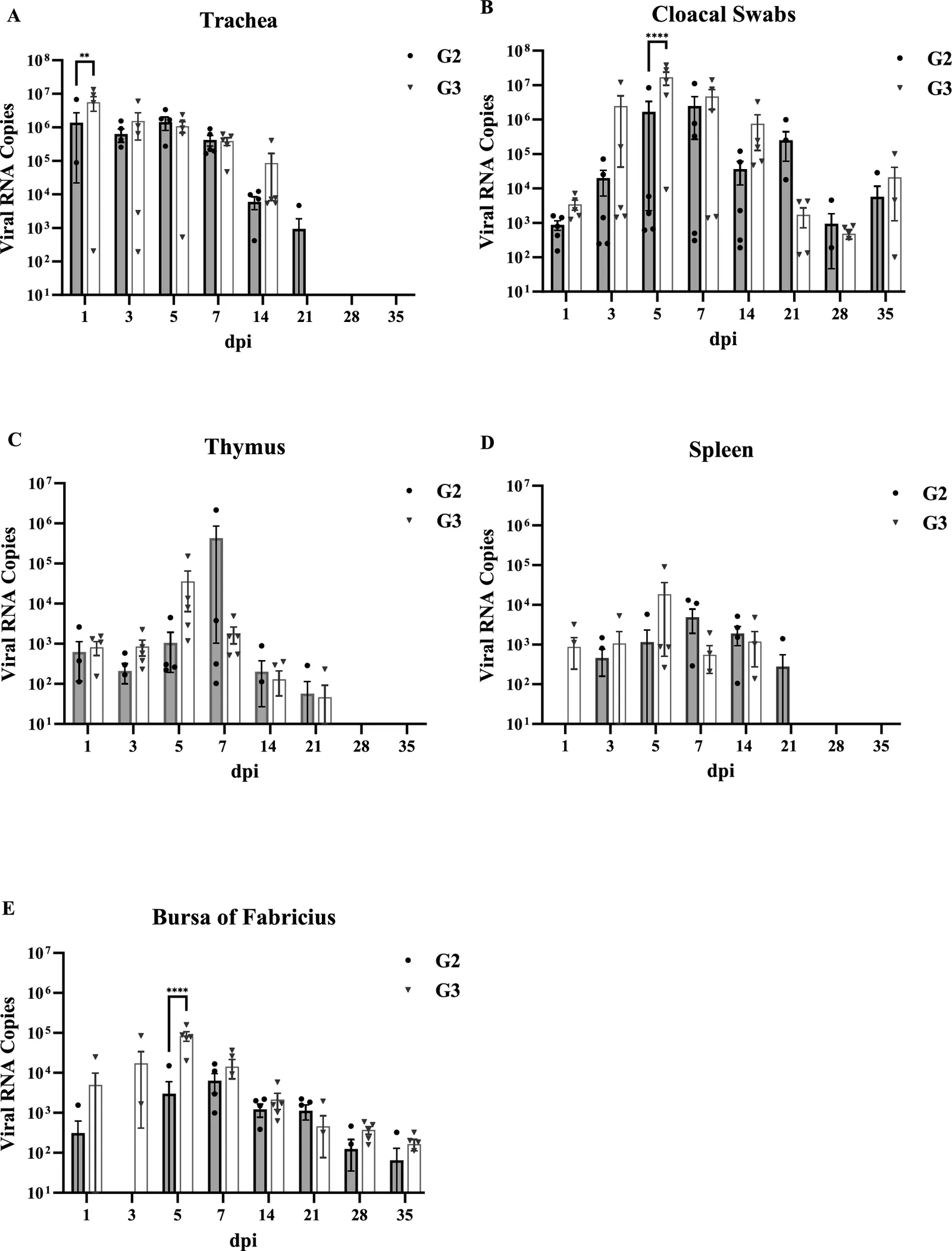
**Viral loads in different tissues in experiment 1** **(A, trachea; B, cloacal swabs; C, thymus; D, spleen; E, BF) of 7-day-old SPF chickens**. The “●” represents the data of G2, the “▼” represents the data of G3, and data not detected are not labeled. Data were analyzed using two-way ANOVA, and bars indicate the mean ± SD.

In the challenged groups of experiment 1, viral RNA remained detectable in cloacal swabs and BFs for up to 35 dpi, while the tracheal, thymic, and splenic tissues tested positive only up to 21 dpi. Tracheal viral loads peaked at 5 dpi, followed by a rapid decline, while the B3-challenged group exhibited significantly higher viral loads compared with the T5-challenged group at 1 dpi (*P* < 0.01). Thymus and spleen samples maintained low viral loads compared with other tissues, except for those from the B3-challenged group at 5 dpi, and thymus samples from the T5-challenged group at 7 dpi. The B3-challenged group exhibited significantly higher viral loads (*P* < 0.0001) in both cloacal swabs and bursal tissues compared with the T5-challenged group at 5 dpi, while peak viral loads in the BF, thymus, and spleen samples were detected at 5 or 7 dpi. No IBV was detected in any tissues harvested from the control groups at any time (Figure 5).

### Histopathology

**Figure 6:**
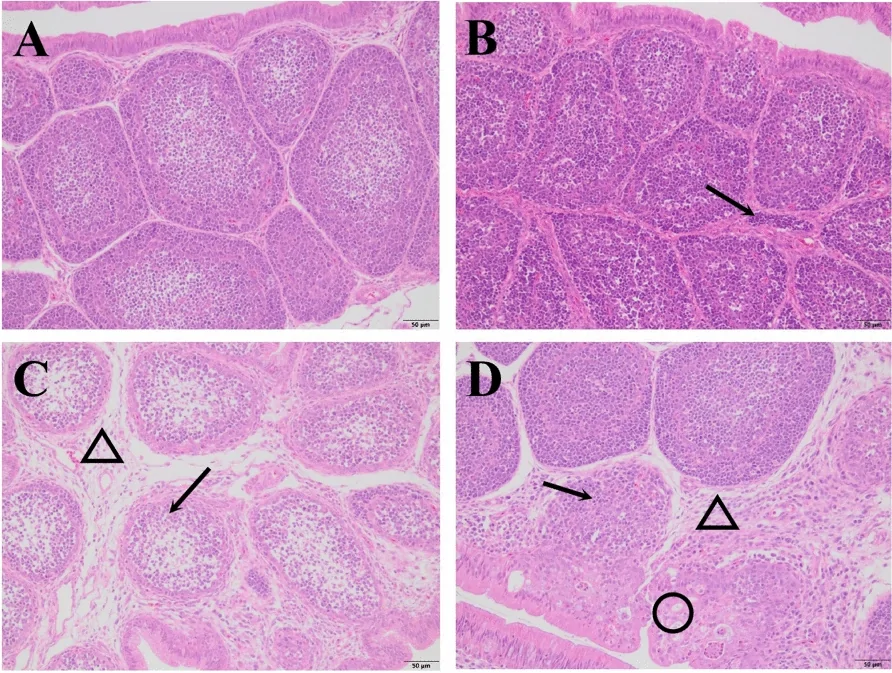
**Histopathologic changes detected in bursae of 7-day-old chicken groups of experiment 1**. **A** Bursae in negative control groups showed no histopathological changes. **B** Atrophy of the lymphoid follicles in the bursae of G3 at 1 dpi (arrow). **C** G2 at 5 dpi showing atrophy and marked depletion of multiple lymphoid follicles (arrow) with lymphocyte infiltration (triangle). **D** G2 at 7 dpi showing squamous cell metaplasia of the lining epithelium and ballooning of mucus cells (circle), atrophy of bursa (arrow) and massive lymphocyte infiltration (triangle).

**Figure 7:**
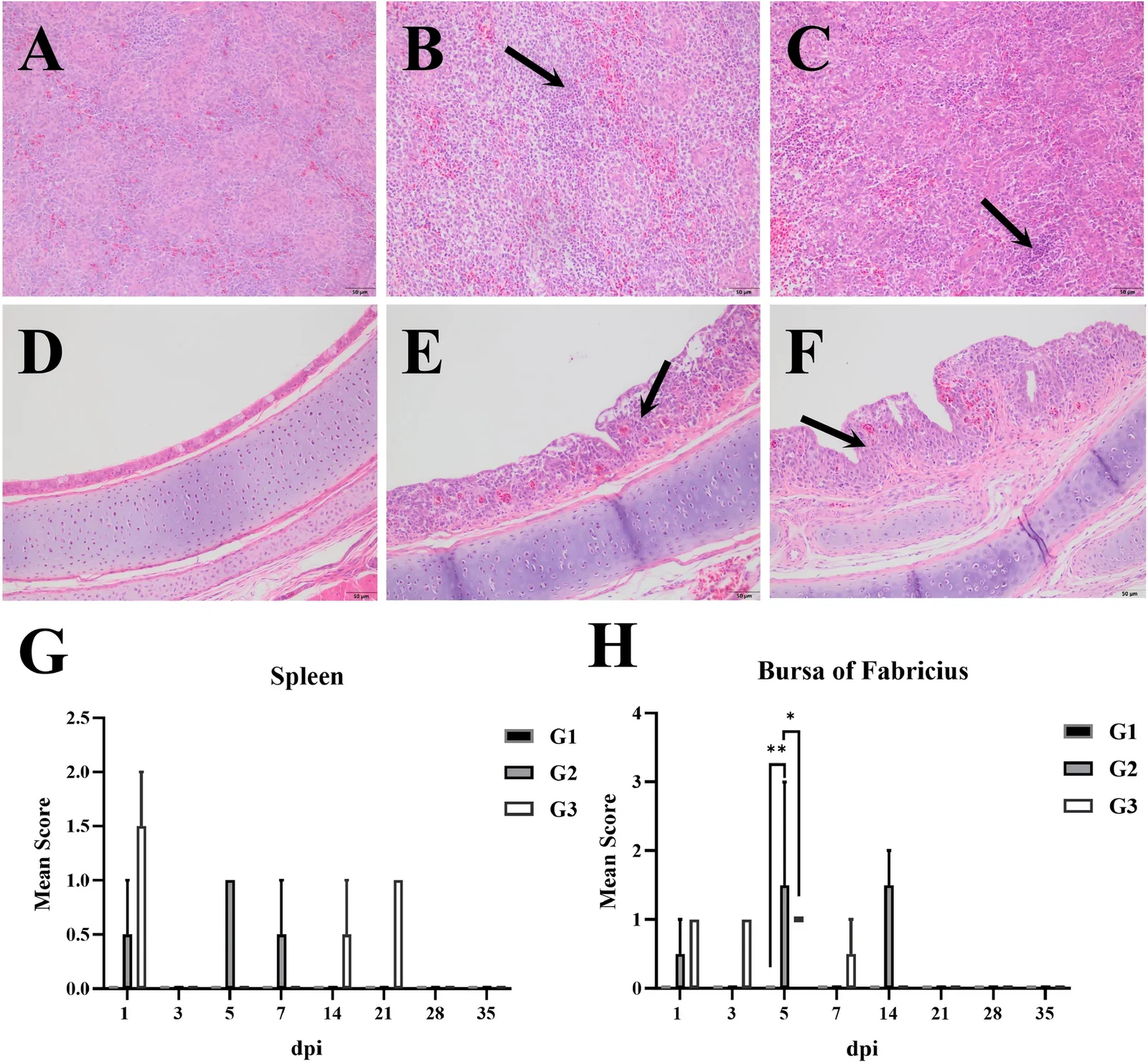
**Histopathologic changes detected in spleens and tracheas of 7-day-old chicken groups, and pathological lesion scores in spleen (G) and** **bursae** **(H) in experiment 1**. **A** The spleen in G1 at 7 dpi. **B** The spleen in G2 at 14 dpi. **C** The spleen in G3 at 1 dpi. **D** The trachea in G1 at 7 dpi. **E** The trachea in G2 at 7 dpi. **F** The trachea in G3 at 7 dpi. Black arrows indicate inflammatory infiltration. Data were analyzed using the Mann–Whitney *U* test for nonparametric data, and bars indicate the mean ± SEM.

The control group showed no histopathological changes (Figures 6A, 7A, D), and no pathological lesions were found in the thymus. In the infected groups of experiment 1, the BFs showed atrophy and depletion of multiple lymphoid follicles (Figure 6B, C), lymphocyte infiltration (Figure 6C, D), and squamous cell metaplasia of the lining epithelium (Figure 6C). Bursal histopathological lesions primarily developed within 14 dpi and were observed as early as 1 dpi in the B3- and T5-challenged groups. Notably, all chickens of B3-challenged group at 5 dpi exhibited bursal pathological lesions, while in the T5-challenged group, lesions occurred sporadically. The BF pathological lesion scores in G2 at 5 dpi were significantly different from those in G1 (*P* < 0.01) and were also significantly higher than those in G3 (*P* < 0.05) (Figure 7H). In the challenged groups, spleen pathological lesions were mainly mild lymphocyte infiltration, and the lesions were similar between the two isolates (Figure 7B, C). There was no significant difference in spleen lesion scores between the challenged and negative control groups (Figure 7G).

In the tracheae of the challenged groups, mucosal epithelial degeneration, hemorrhage, and lymphocyte infiltration were the main causes of lesions (Figure 7E, F), while the tracheae of the B3-challenged group were more severely affected than the T5-challenged group.

### Immunohistochemistry

**Figure 8:**
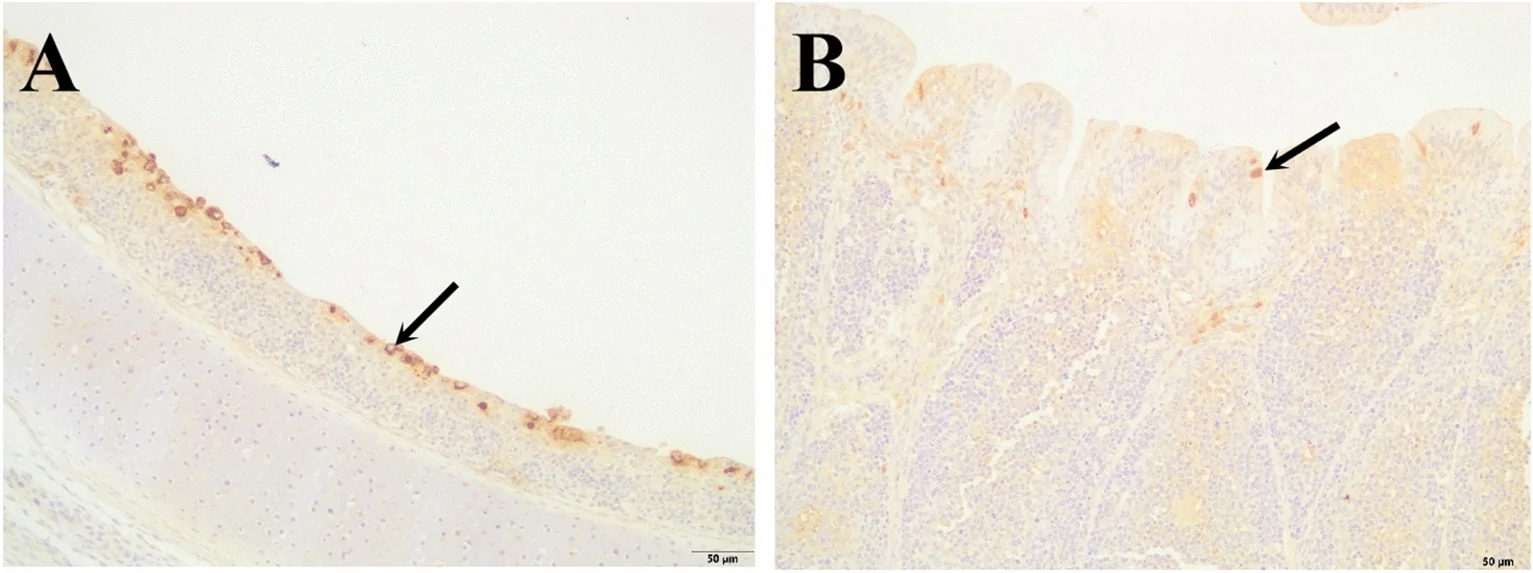
**IBV antigen detection in tissues of SPF chickens in infected groups of experiment 1 by immunohistochemistry (IHC): IBV IHC staining in the trachea (A)** **and bursae** **(B)**. Black arrows indicate IBV antigen.

The tissues of the negative control group did not exhibit any immunopositive reaction against the IBV antigen. In the infected groups, the immunopositive reaction against IBV was only detected in the trachea (Figure 8A) and BF (Figure 8B), as early as 3 dpi. Immune reactivity was confined to the epithelial cells, and no IBV antigens were observed in the spleen or thymus.

### TUNEL analysis

**Figure 9:**
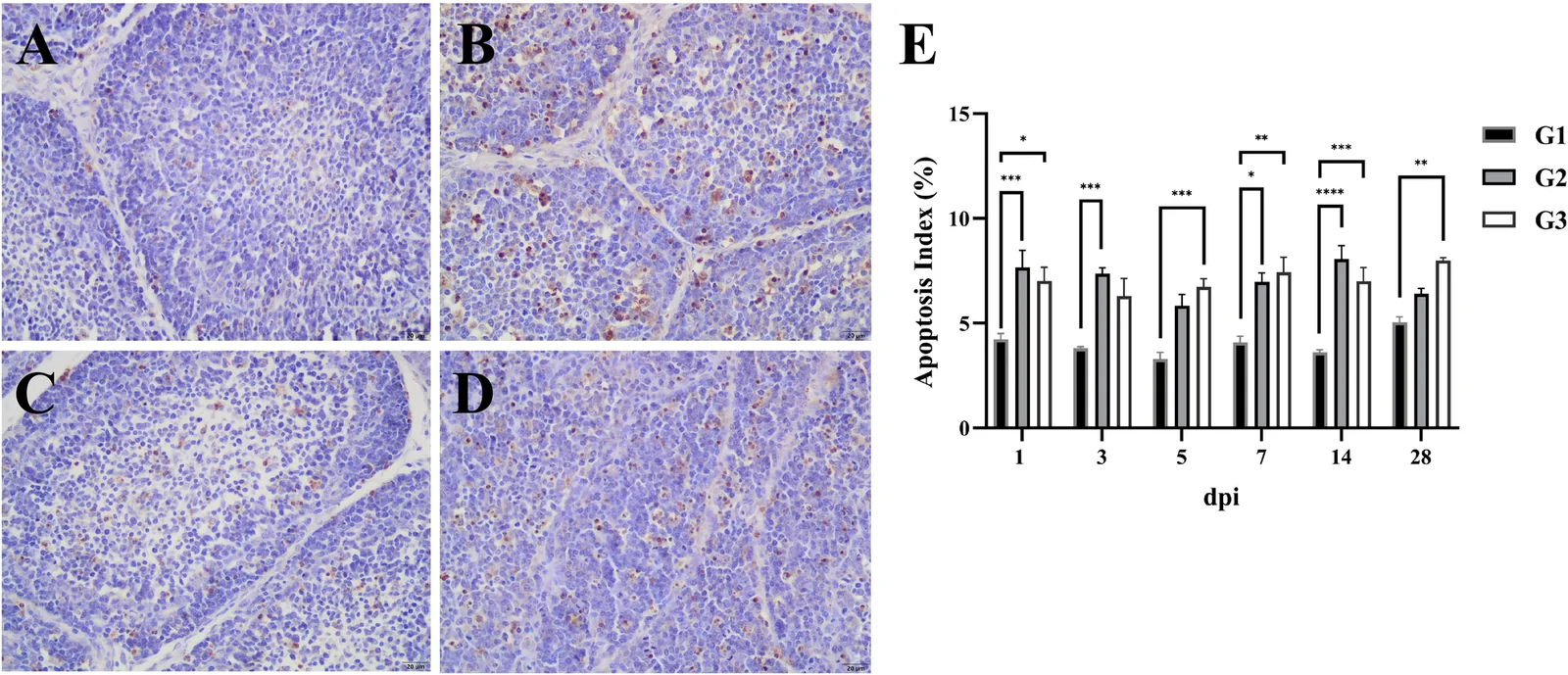
**Apoptosis induction in the BF following IBV infection in 7-day-old chickens** **of experiment 1**. TUNEL-positive apoptotic cells are stained brown. **A** BF of G1 at 14 dpi. **B** BF of G2 at 14 dpi. **C** BF of G1 at 7 dpi. **D** BF of G3 at 7 dpi. **E** Apoptotis index in BF. Data were analyzed using the Kruskal–Wallis test for nonparametric data, and bars indicate the mean ± SEM.

Bursal tissue sections from negative control and infected groups were evaluated via TUNEL assay (Figure 9). TUNEL staining of negative bursal tissues revealed only a small number of apoptotic cells, whereas the apoptotic cells in the challenged groups were randomly distributed. Compared with the negative control group, the AI in BF was significantly increased in the challenged groups following IBV infection (Figure 9E). Notably, B lymphocytes in the T5-challenged group showed a significant increase in apoptosis at 1, 3, 7, and 14 dpi, with *P*-values ranging from *P* < 0.05 to *P* < 0.0001 (Figure 9B, E). A consistent trend was observed in the B3-challenged group, where apoptosis levels were significantly higher at 1, 5, 7, 14, and 28 dpi, with statistical significance ranging from *P* < 0.05 to *P* < 0.001 (Figure 9D, E). No significant difference in AI was observed between the T5- and B3-challenged groups.

## Discussion

This study identified two IBV strains, the CK/CH/FJ-B3/2023 strain (bursal origin) and the CK/CH/FJ-T5/2023 strain (tracheal origin), that clustered with other GI-19 strains and exhibited recombination events with the 4/91 vaccine strain. While present in the thymus, spleen, and BF, the two isolates showed bursa-specific tropism and depletion of the central lymphoid follicle. Infection of 7-day-old SPF chicks led to prolonged detection of viral nucleic acids and histopathological atrophy in the BF accompanied with cell apoptosis and elevated levels of proinflammatory cytokines (IFN-*γ*, TNF-*α*, IL-1*β*, and IL-6), and ultimately leading to humoral immunosuppression.

The GI-19 lineage of IBV is presently widespread across Asia [33], Europe [34], and South Africa [35]. This lineage is known to evolve, recombine, and form independent clades in different countries, where the overall evolutionary rate was estimated at approximately 10^–3^ to 10^–4^ [36]. Viral recombination events were detected between field and vaccine strains [37], and this study showed that the two GI-19 isolates isolated from flocks vaccinated by multiple activated vaccines exhibited recombination events with the 4/91 vaccine, which is consistent with other reports [38, 39]. Introduction of the 4/91 vaccine could have driven genetic changes in some viruses, showing its ability to interact with field viruses [40, 41]. Compared with the T5 strain, the full-length genome of the B3 strain contains an additional recombinant fragment located at positions 1 to 976 at the 5′ end, which is derived from the 4/91 vaccine strain. This fragment was located within the nonstructural protein region (Nsp2 rather than Nsp1), whereas *γ*-CoVs lack this protein [42], which may serve as a key virulence-related factor in the IBV genome [43]. Additionally, it has been reported that 4/91-like vaccine-derived strains can still be detected in chicken flocks not vaccinated with the 4/91 vaccine [44], and GI-13 strains continued to circulate despite cessation of their use [45]. This may have contributed to the high positive rate of viral nucleic acid detection in the BF at 35 dpi; therefore, continuous monitoring of circulating viruses is necessary.

Infectious bronchitis virus initially colonizes the upper respiratory tract and then disseminates to other tissues for further replication, resulting in broader tissue tropism [3, 11]. An excessive focus on the obvious clinical manifestations of IBV may result in the neglect of potential damage to other tissues. For example, the enterotropic IBV can replicate in the oviduct epithelium and is associated with a decline in egg production [46]. During clinical sampling, the flocks from which the two IBV strains were isolated primarily exhibited respiratory distress. Autopsy examinations identified bursal atrophy—initially misattributed to IBDV [47]—but subsequently molecularly identified and experimentally confirmed as IBV. These findings emphasize the necessity of expanding diagnostic sampling beyond respiratory tissues, as viral persistence in nonrespiratory tissues may significantly impact pathogenesis and poultry production. Furthermore, viral isolation from immune organs or tissues other than the trachea is initially difficult. The enterotropic isolate G and the B3 strain (bursal origin) required five egg passages for successful isolation [4], while the T5 strain required only three passages. This variation is most likely due to the nature of the guts or bursa, where lymphocytes mount immune responses to foreign antigens. It remains to be further explored whether there are pathogenicity differences among IBV isolates recovered from different tissues of the same chicken flock.

The GI-19 lineage of isolates is mostly responsible for respiratory infections and nephritis [48], while the QX strains are mostly detected in the trachea and kidneys [34]. Reports suggest that tests are positive in the BF by 14 dpi following a single infection [4, 27], with a maximum duration of up to 21 dpi, or 28 dpi in the case of IBDV coinfection [29]. This study showed that, for these two IBV strains, detection time can be extended to 35 dpi in BFs and cloacal swabs. This is possibly due to IBV clearance delay during single infections, resulting in delayed serum antibody seroconversion at 14 dpi. Additionally, IBV could also be detected in the spleen, which is consistent with previous reports of IBV antigen presence in both chicken embryos or chicks [48, 49], where tests returned positive by 21 dpi. Notably, the T5 isolate exhibited significantly longer persistence in the trachea and spleen up to 21 dpi, accompanied by a lower ‌IFN-*γ* levels in the T5-challenged group compared with the B3-challenged group beyond 14 dpi. As reported for severe acute respiratory syndrome coronavirus 2 (SARS-CoV-2), ‌IFN-*γ* possesses unique immunoregulatory activities that are critical for the host innate defense against microbial infections, and it also plays a role in mediating antiviral protection, particularly in the long-term control of persistent viral infections [50]. IFN-*γ* could also enhance the ability of host against IBV [51], and this may provide a plausible explanation for the extended long-term viral persistence observed in the trachea and spleen of the T5-challenge group, where relatively low levels of ‌IFN-*γ* were detected.

Histopathological lesions in the BF are mainly attributed to IBDV infection, accompanied by atrophic bursae and depleted lymphoid cells [47]. While present in the thymus, spleen, and BF, the two IBV isolates showed bursa-specific tropism, with depletion of the central lymphoid follicle. This is supported by previous reports on different genotypes or serotypes of IBV [15, 27, 52, 53]. Interestingly, the emergence of pathological bursal lesions coincided with the detection of viral nucleic acids in the bursae within 7 dpi. Consistently, no viral nucleic acids were detected in any bursal tissue samples that showed no observable pathological damage at the same time points. Additionally, IBV is an epitheliotropic virus that can be detected as early as 16 h post-infection [54], although the onset of pathological lesions exhibits strain-dependent variation. Specifically, the B3 strain (bursa-derived) demonstrated lesions as early as 1 dpi, while the DMV/1639 and T5 strains showed lesions at 3 and 5 dpi, respectively [15]. Differing from IBDV infection, which causes more severe and consistent BF damage [32, 55], a bursa with a BBIX < 0.70 [BBIX = (bursa:BW ratios in the infected group)/(bursa:BW ratios in the control group)] combined with pathological lesion was considered as morphological atrophy [56]. In the present study, the two IBV isolates displayed mild and heterogeneous pathological lesions. The BBIX value at 28 dpi in the B3-challenged group was 0.71 with no bursal pathological lesions, indicating a lower degree of virulence of two isolates and a more variable host response compared with IBDV strains. Moreover, significant reductions in body weight were observed in the B3-challenged group compared with the negative control group at both 14 and 35 dpi. A parallel decrease in bursa weight was noted at 35 dpi. The relative bursa weight in the T5- and B3-challenged groups showed no significant difference compared with the negative control, since some bursae were enlarged while others were atrophied, resulting in an overall average that masked individual pathological variations. Also, the spleen relative weight in the B3-challenged group showed a significant decline at both 3 and 5 dpi, accompanied by mild lymphocyte infiltration. This confirms that IBV induces significant bursal and minor splenic lesions, contradicting earlier findings [15].

Despite current research, the characteristics of IBV immunosuppression are not clear. Pope [57] proposed four methods to assess immunosuppression, one of which includes the recognition of a marked reduction in lymphocytes in both the primary and secondary lymphoid organs. Although IBV has been isolated from the HG [58], and pathological lesions have been reported in lymphoid tissues [15], evidence for immunosuppression is limited, especially with interference from HI titers of other vaccines administered during IBV infection [59]. Abnormal changes seen in lymphoid tissues may also be caused by clinical aspects with resultant lesions, such as stress and nutrition [60], and a multidisciplined investigative approach is required to determine the cause [57]. In this study, NDV HI antibody detection was conducted in addition to cytokines proteins and bursal apoptosis analyses to further confirm whether IBV induces immunosuppression. Results showed that a delay of NDV HI titers was observed in chicks with IBV infection, especially in B3- and T5-challenged groups at 14 dpi and in the B3-challenged group at 28 dpi compared with the NDV immune group. This indicates that IBV infection interferes with humoral antibody production in a manner similar to IBDV infection, which referred to the humoral immunosuppression evaluation paradigm [61]. In addition, significantly higher levels of TNF-*α* and IL-1*β* were induced by these two IBV strains, which are involved in recruiting polymorphonuclear and mononuclear leukocytes to the primary infection site [62]. This cytokine pattern differs from previously reported IBV infection profiles, which decline at 8 dpi [63], but resembles the cytokine trends and bursal atrophy observed in IBDV VP2 infection [64]. These cytokines remained elevated until 28 dpi, suggesting potential excessive immune activation and delayed humoral responses to NDV vaccination. However, detectable bursal pathological lesions in the BFs of IBV-infected chicks were restricted to the period before 14 dpi, rather than the permanent bursal damage induced by IBDV infection. This is consistent with the detection of viral nucleic acid in the bursae at 35 dpi, raising the hypothesis that the residual low-level viral nucleic acid may continuously induce cytokine production. Furthermore, the upregulation of ‌IFN-*γ* and IL-6 observed in this study may promote immune dysregulation and exacerbate tissue damage [25, 63]. Notably, IBDV destroys B cells, triggering not only severe necrosis and lymphoid depletion but also extensive apoptosis [65], which ultimately leads to persistent humoral immunosuppression [66, 67]. Previous studies have demonstrated a strong correlation between apoptosis in tracheal organ cultures (TOCs) or renal cells and the capacity of IBV strains to induce renal or respiratory lesions in chickens [68, 69]. Consistent with these findings, this study confirmed that bursal cell apoptosis is associated with the formation of pathological lesions before 14 dpi, while the mechanism underlying sustained apoptosis up to 28 dpi remains to be further elucidated. Furthermore, the low viral nucleic acid levels in the BF at 28 and 35 dpi may represent residual nonreplicating viral nucleic acid fragments, and no viral nucleic acids were detectable in the spleen or thymus. Consistent with this observation, bursal function was found to fully recover at 35 dpi. Collectively, these results demonstrate that IBV-induced humoral immunosuppression may represent a transient state of immune dysfunction rather than the permanent impairment caused by IBDV infection. Notably, the current evaluation indicators for immunosuppression are mainly focused on humoral immune response, cellular apoptosis, and cytokines, while functional verification of key cellular subsets (such as T cell subpopulations and bursal B cell progenitor pools) remains to be addressed in future studies.

## Conclusions

Our study systematically characterized the humoral immunosuppression induced by two IBV isolates, with a particular focus on bursal apoptosis and blood cytokines. Whole-genome analysis confirmed that the two IBV isolates, CK/CH/FJ-B3/2023 (bursal origin) and the CK/CH/FJ-T5/2023 (tracheal origin), share recombination events with the 4/91 vaccine strain. Prolonged viral detection persisted for 35 dpi via the cloaca and bursae, with histological evidence of bursa-specific tropism characterized by central lymphoid follicle depletion confirmed by immunohistochemistry. Seroconversion was delayed until 14 dpi and was accompanied by reduced bursa relative weight in infected groups. When combined with inactivated Newcastle disease vaccination, both IBV isolates induced significant humoral immunosuppression at 14 and 28 dpi, with full recovery of antibody production at 35 dpi. Notably, both isolates triggered sustained proinflammatory cytokines responses, including ‌IFN-*γ*, TNF-*α*, IL-1*β*, and IL-6, which persisted up to 28 dpi, alongside significantly increased levels of bursal cell apoptosis across the observation period. Therefore, these findings highlight the humoral immunosuppressive capacity of these recombinant IBV variants and provide evidence for the continuously evolving pathogenicity of circulating IBV strains in China.

## Supplementary information

**Additional file 1. A total of 78 reference strains isolated from flocks.**

## Data Availability

All data generated during this study are included in this published article.
