# Supplementary material for: Recombinant GI-19 infectious bronchitis virus induction of humoral immunosuppression with reduced HI antibody response following Newcastle disease virus vaccination and bursal cell apoptosis
Source: Vet Res. 2026 Sep 24;57:170. doi: 10.1186/s13567-026-01839-2 (PMC13602560; doi:10.1186/s13567-026-01839-2)
Supplement: Supplementary file 1 [file 13567_2026_1839_MOESM1_ESM.docx]

**Additional file 1** 78 references strains isolated from flocks

| IBV strain | Genotype | Accession  number | IBV strain | Genotype | Accession  number |
| --- | --- | --- | --- | --- | --- |
| Beaudette | GI-1 | M95169.1 | Qu mv | GI-20 | AF349621.1 |
| H120 | GI-1 | FJ888351.1 | CU83148 | GI-20 | AF391158.1 |
| CK/CH/LLN/111169 | GI-1 | KF411040.1 | Spain/97/314 | GI-21 | DQ064806.1 |
| Holte | GI-2 | GU393336.1 | Spain/98/313 | GI-21 | DQ064808.1 |
| Guangzhou-06 | GI-2 | GQ844988.1 | 66GD-98VI | GI-22 | KC577397.1 |
| Grey | GI-3 | L14069.1 | 40GDGZ-97I | GI-22 | KC577382.1 |
| JMK | GI-3 | L14070.1 | variant 2 | GI-23 | AF093796.1 |
| Holte | GI-4 | L18988.1 | Eg/CLEVB-2/IBV/012 | GI-23 | JX173488.1 |
| 56GX-98I | GI-4 | KC577394.1 | V13 | GI-24 | KF757447.1 |
| N1/62 | GI-5 | U29522.1 | V24 | GI-24 | KF757450.1 |
| HN99 | GI-5 | AY775551.1 | CA/1737/04 | GI-25 | EU925393.1 |
| V5/90 | GI-6 | U29520.1 | DMV/5642/06 | GI-25 | EU694402.1 |
| TP/64 | GI-7 | AY606320.1 | NGA/B401/2006 | GI-26 | FN182243.1 |
| ck/CH/LHLJ/110664 | GI-7 | JQ739299.1 | NGA/BP61/2007 | GI-26 | FN182268.1 |
| L165 | GI-8 | JQ964061.1 | Georgia 08 | GI-27 | GU301925.1 |
| L613 | GI-8 | JQ964066.1 | GPL8225 | GI-27 | GU437858.1 |
| ARK99 | GI-9 | M99482.1 | ck/CH/LGX/111119 | GI-28 | KX640829.1 |
| 54HaN-95I | GI-9 | KC577392.1 | GX-NN-13 | GI-28 | JX291989.1 |
| B | GI-10 | AF151954.1 | gammaCoV/ck/China/I0118/14 | GI-29 | KY407558.1 |
| T6 | GI-10 | AF151960.1 | gammaCoV/ck/China/I0111/14 | GI-29 | KY407557.1 |
| UFMG/G | GI-11 | JX182775.1 | Mex-07-1 | GI-30 | OR268746.1 |
| IBV/Brasil/351/1984 | GI-11 | GU393339.1 | Mex-1 | GI-30 | OR268745.1 |
| D3896 | GI-12 | X52084.1 | strain V1397 | GII | M21968.1 |
| NGA/295/2006 | GI-12 | FN182276.1 | D1466 | GII | M21971.1 |
| 4/91 | GI-13 | AF093794.1 | V18/91 | GIII | U29521.1 |
| Moroccan-G/83 | GI-13 | EU914938.1 | V6-92 | GIII | DQ490219.1 |
| UK/7/91 | GI-13 | Z83975.1 | DE/072/92 | GIV | U77298.1 |
| Belgian isolate B1648 | GI-14 | X87238.1 | GA/Avial1 | GIV | EU283066.1 |
| NGA/324/2006 | GI-14 | FN182277.1 | GA/13055/00 | GIV | AF338719.1 |
| B4 | GI-15 | FJ807932.1 | N1/03 | GV | FJ235194.1 |
| K281-01 | GI-15 | AY257062.1 | N4/03 | GV | DQ059620.1 |
| IZO 28/86 | GI-16 | KJ941019.1 | SDIB781/2012 | GVI | KF007209.1 |
| 2992/02 | GI-16 | AY606323.1 | GX-NN09032 | GVI | JX292013.1 |
| CA/Machado/88 | GI-17 | AF419315.1 | gammaCoV/ck/China/I0636/16 | GVII | MH397183.1 |
| CV-56b | GI-17 | AF027509.1 | GX-NN130021 | GVII | KM365468.1 |
| JP8127 | GI-18 | AY296744.1 | CK/DE/IB80/2016 | GVIII | MT591566.1 |
| 48SD-96VI | GI-18 | KC577388.1 | gCoV/ck/Netherlands/D2860/2019 | GVIII | OR791595.1 |
| LX4 | GI-19 | AY189157.1 | Mex-56-7 | GIX | OR268750.1 |
| 58HeN-93II | GI-19 | KC577395.1 | Mex-14P | GIX | OR268749.1 |
